# Small Pox as It Appeared in Richmond, Va., and Its Vicinity in the Winter of 1855 and 1856

**Published:** 1856-06

**Authors:** Thomas Pollard

**Affiliations:** Richmond, Va.


ATLANTA

Medical and Surgical Journal.

Vol. I.] JUNE, 1856. [No. 10.

### ORIGINAL COMMUNICATIONS.

### ARTICLE I.

*Small Pox, as it appeared in Richmond, Va., and its vicinity in the winter of* 1855 *and* 1856. By Thomas Pollard, M. D., of Richmond, Va.

Small Pox, as an epidemic, has been quite prevalent in our city during the past winter, and also in some of the adjoining counties. Its fatality has not been great, most of the cases having been of modified form. In our hospital, the admissions have been one hundred and the deaths about ten.

One of the striking features of the epidemic has been, the number of cases of varioloid, occurring after vaccination, a feature unfortunately not new in the modern history of the disease. Some severe cases of varioloid have taken place under my observation, after vaccination practiced a number of years since, generally before puberty, and in the "City Small Pox Hospital," where I have been kindly invited by the physician of the establishment, Dr. A. Snead, to examine the cases, it has been almost always found that in the decided cases of varioloid, vaccination had been practiced before the growth of the patient. This coincides with the opinion of Dr. Gregory, of the London Small Pox Hospital, who says that modified small pox after vaccination, is most apt to occur between the ages of fifteen and twenty-one, and that it is not necessary to re-vaccinate before the tenth year. Unfortunately, Dr. Gregory has modified his opinion still further, and now says that vaccination, after the fifteenth year, does no good, not being protective against small pox, and is in favor of returning to inoculation. Fifteen years after vaccination was generally adopted, varioloid after vaccination became quite prevalent. Indeed, Dr. Gregory says, he had not seen it to occur before this period. This again became more manifest in the epidemics in Scotland, in 1818, (described by Thomson,) and in France, in 1825 and 1828, and has been very striking in the great epidemics afterwards occurring in France, Denmark, and Germany. Jenner, who lived twenty years after the full introduction of vaccination, lived to see that vaccination was not so perfect a protection as he once so fondly hoped, and accounted for it upon the supposition that vaccination was rendered imperfect, 1st. By the existence on the skin of chronic cutaneous eruptions; 2d. The use of spurious vaccine matter; 3d. By depriving the vaccine vesicle of its lymph, or injuring it by external violence so as to give rise to common phlegmnous inflammation. His faith in the protective power of vaccination was so great that he did not believe its effects could wear out of the system. Later observation and experience, we think, has demonstrated that the effects of vaccination are impaired, and in some cases extinguished by time.

As early as 1804, eight years after the inauguration of vaccination, Dr. Goldson expressed his doubts of the permanency of vaccination, but these doubts were silenced by Jenner, who inoculated three persons who had had cow-pox twenty-three, twenty-seven, and thirty years before, without effect. This experiment proved that these three persons were in all probability not liable to have small pox, either through their previous protection, or from want of susceptibility of the system at that particular time, no more.

Dr. Watson says, "it is certain that in many if not in all of those who have gone through the vaccine disease, revaccination at a distant period produces, in a greater or less degree, its primary effects." In the midst of extensive epidemics of small pox in later times, in Germany, France and Denmark, it was observed that the number of varioloid cases continued to increase, and those were affected with it who had been vaccinated for fifteen, twenty, and twenty-five years, while those escaped who had been vaccinated for a few months or years, (Dictionnaire de Medecine—Revue Medicale, January, 1829.) An account of the epidemics observed in 1828 and in 1829, at Marsailles, Beaucaire, and at several other places in France, furnished a great number of like facts. Thence re-vaccination was proposed as a means to remedy this evil, though some medical men in France had already vaccinated with success. On the continent generally, it soon came to be tried on great numbers. In Prussia, it was adopted as a general measure in 1833. The success was so decided as to induce the government to continue the practice, which was extended also to the army. The returns soon exhibited less of varioloid and many cases of successful re-vaccination. In 1833, the operation was tried on 48,478 persons, with success in 15,269. In 1834, re-vaccination was practiced on 33,454, of which 16,679 succeeded. In 1835, 39,192 were re-vaccinated, of which 15,315 were successful. In 1836, re-vaccination was performed on 42,124, with success in 18,136. In the same year, Dr. Aggeno re-vaccinated 962 persons, with success in 822. In 1836, in the Danish army, 2,756 subjects were re-vaccinated with success, the operation having no effect at all in 1,208. In 1839, in the Prussian army, 41,481 soldiers were re-vaccinated; of this number, the vaccine cicatrices were apparent in 33,225, slightly distinct in 5,889, and not apparent at all in 2,367. The re-vaccination succeeded in 19,249, and produced an irregular eruption in 8,534.

Dr. Mingenthal (Gaz. Medical, 1843,) re-vaccinated 1810 adults, of whom, 1780 had been vaccinated in their infancy. Of these, 1,163 had complete success. In the army of Hanover, in the years 1837, 1838, and 1839, in 9,366 re-vaccinations, 1,068 succeeded completely.

In France, the operation has not been practiced so extensively. The "Royal Academy of Medicine," long consulted by the Minister, upon the necessity of re-vaccination, no doubt with the dread of underrating vaccination staring them in the face, decided against it. Some of the most celebrated medical men of France have protested against this decision, and reported at the same time numerous cases of success of re-vaccination in their practice. Dr. Hardy, in an extended essay, exhibits the fact, that the documents collected in England, bearing on this point, agree with those furnished by the medical men of Denmark, Prussia, Sweeden, Germany, and Italy.

Dr. Villaret (Gaz. Med., 15 Oct., 1843,) publishes the results of re-vaccinations practiced in the 7th Regiment of Dragoons. In 401 subjects, re-vaccinations succeeded 307 times—the operation succeeded 97 times in 153 persons, having previously had varioloid. In another series of cases, he found re-vaccination to succeed in 402 cases out of 447, and in 123 who had had varioloid, 83 re-vaccinations were successful. The "Academy of Sciences of Paris," in 1846, published their report on this subject. Their conclusions, in a condensed form, are as follows: 1st. The preservative virtue of vaccination for the greatest number of those vaccinated is absolute, and temporary for a small number; in the last, indeed, it is almost absolute up to adolescence. 2d. Variola rarely affects the vaccinated before the age of ten or twelve years; it is from this epoch up to thirty and thirty-five years that they are principally exposed. 3d. Besides its preservative effect, vaccination modifies and makes mild the symptoms of variola. 4th. The local intensity does not appear intimately allied with the preservative virtue of vaccination; nevertheless to preserve the properties of vaccine it is prudent to renew it as often as possible. 5th. Among the means proposed for its renovation, the only one in which science can repose confidence up to this time consists in taking vaccine from its source. 6th. Re-vaccination is the only means of proof that science possesses to distinguish the vaccinated who are absolutely protected from those who are protected but partially. 7th. The test of re-vaccination does not constitute a certain proof that those with whom it succeeds were destined to have variola, but only a sufficiently great probability that it is particularly among those that the disease is susceptible of being developed. In ordinary times, re-vaccination ought to be practiced after the fourteenth year; in times of epidemics, it is prudent to shorten this period. (Dictionnaire de Medecine Art.; Vaccine, Guer. Sant. et Blache.) We have been at the pains to record these rules because we think the profession can follow none better for their guide on this subject. We believe them, as far as the present condition of vaccination can instruct us, to be true. The proposition laid down in No. 7 is doubtless correct, and the success of re-vaccination is not absolute proof that the individual would contract small pox or varioloid if exposed to the contagion, but the presumption and the evidence is certainly strong, that where re-vaccination succeeds so would small pox there find a nidus. Thus much has been devoted to this subject, because we feel its importance, and desire that the profession should be united on it. We think the evidence is sufficient to convince every one that the preservative effect of vaccination is weakened by time and that it is our duty to advise, and propagate with zeal the doctrine of re-vaccination. We should teach ourselves and our friends and constituents its true value, and by abstaining from re-vaccinations we permit the spread ot varioloid, and in some cases of variola, which will do more to impair confidence in vaccination than anything else. We would not detract a particle from this great boon to mankind, but at the same time we should not rely blindly upon its perpetual and unceasing protection.

Our observation, and we believe that of the medical men in this city generally, during the past epidemic, was in favor of the still active property of the vaccine virus, and that it has not been seriously impaired by time and transmission through the human system. During the epidemic there has been great susceptibility to the vaccine disease, and at another time we might feel possibly more that the virus has been impaired by time. On this subject, Dr. Watson remarks, that from close observation while senior Censor of the College of Physicians at the National Vaccine Board, "he was satisfied that lymph which had been transmitted from person to person from the times of Jenner, was capable of producing as perfect a cow-pox vesicle as at first; and as Dr. Heim asserts, there are no less than five spurious kinds of cow-pox, all communicable by inoculation, from the teats of the cow to the human body, I cannot help thinking recourse should not be had easily or heedlessly to lymph recently obtained from the cow." Particularly should this advice be heeded, if a statement made by Wilson (Diseases of the Skin, p. 144) is true. He says, "it has been observed that when the epizootic disease presents symptoms of great severity, the effects produced on man by inoculation from such cases, were also severe, and often serious, contrasting strongly with the mild affection engendered by the virus from the ordinary discreet form of cow-pox. Mr. Macpherson, in experiments with this virus, in Bengal, in 1837, found that an eruption was developed which was identical with small pox. Mr. Wood, of Gowalpara, in 1839, met with similar cases of so great severity, that he was led to contemplate the institution of inoculation with small pox virus, as a safer expedient. At Silhet, Mr. Brown removed some dark colored scabs from a cow laboring under variolous disease, and inoculated several children with them. These cases exhibited nothing remarkable, excepting a somewhat greater degree of constitutional disturbance on the 8th day than usual. After two months children inoculated from this stock were attacked on the 8th day with severe fever, followed by an eruption which spread over the whole body, and in one case proved fatal." Others take a different view, (we have quoted the opinion of the "Academy of Sciences" on this question,) and attribute some of the defects of vaccination to virus weakened by long transmission. Virus has been procured from the cow, affected with the natural vaccine disease, and among others, by Mr. Easthin, of Gloucestershire; Mr. Fox, and Mr. Sweeting, of Dorsetshire; and Mr. Ceely, of Buckinghamshire; and also, by Mr. Saunoy, of France. (Wilson on the Skin.) Virus thus obtained, has been transmitted, and brought into general use. The inoculation of the cow, as a means of obtaining fresh virus, seems of very uncertain character. Many experiments have been made, and as far as I have been able to ascertain, with only two or three positive results. Dr. Basil Shiele, of Khasan, is said to have succeeded several times in inoculating the udder of cows. (Wilson.) Dr. Carpenter, of Philadelphia, also says he has succeeded. Richter asserts, on the authority of Gassner, that he has been successful; and Dr. Ozaman, of Lyons, states he has demonstrated the identity of the two matters, by mixing variolous matter with fresh cow's milk, and by its use, producing genuine vaccine disease. (Eberle, p. 453.) The experiment was recently performed in this city, at the Small Pox Hospital, by Dr. James Bolton, in presence of several medical men, without success; though the experiment was repeated, and the cow subjected in her stall to the fomites of the rejected clothing, and excretions of the patients in the hospital. Dr. Wilson (on the Skin) remarks, that the cow-pox virus immediately from the cow, takes with difficulty on the human; and so the small pox virus from the human with difficulty acts upon the cow. From the small number of cases recorded of successful inoculation of the cow, it is doubtful whether the experiment has really ever succeeded. Guersant and Blache (Dictionnaire de Medicine) say it has never succeeded; though there can be little doubt of the original identity of the two. There seems scarcely a doubt of the small pox attacking the cow in the natural way, and producing what we call cow-pox virus; (see Wilson on art. "Variola Vaccine;") and there is very good evidence to believe that other animals are subject to small pox contagion. Mr. Howell, a gentleman of respectable rank and character, who resided long in India, remarks: "In an epidemic season of the small pox, turkeys, chittygong fowls, madras capons and other poultry, are carried off in great numbers by the disease, and have the symptoms usually accompanying every stage of the distemper. I had a favorite parrot who died of it in 1774. In him I had a fair opportunity of observing the regular progress of the disorder. He sickened, and had an ardent fever full two days before the eruption, and died on the seventh day of the eruption." (American Med. Rec., Jan. 1829.)

Analogy in other diseases would support the same doctrine. We know glanders to be communicated by the horse to the human, rabies canina to the human; and we have good reason to believe that cholera sometimes attacks animals.

Varicella prevailed to some extent in this city during the prevalence of small pox. Nothing occurred as far as we are informed, to change the opinion now generally entertained among medical men, of the diversity of the two diseases. We say generally entertained opinion, for there are still some medical men of eminence, both in this country and in Europe, who believe varicella and variola to be one and the same. A few facts, such as have come under the view of almost every city practitioner, are sufficient to outweigh all the ingenious reasoning of Thomson on this subject, whose whole book is devoted to an attempt to prove their identity. We, for example, have seen recently chicken-pox occur in a house among unvaccinated children without producing any bad effects. We have seen also vaccination succeed perfectly in two cases, of persons who, three years since, had chicken-pox. The diagnosis generally laid down between these diseases, seems supported by close observation—the commencement of the eruption of varicella is upon the head, whereas variola begins on the face—and the vesicular character of varicella, from the first, is very aptly compared to effects produced by drops of boiling water falling on the surface.

In the city some, and in the neighboring counties many, of the cases, both of variola and varioloid have been the result of vaccination. Whence the virus was obtained producing these results, has been a matter of discussion, and what its peculiar nature, has been a subject of more interesting enquiry. Most, if not all, of this virus seems to have been distributed by the state vaccine agent, resident in Richmond, and by one of our most popular medical practitioners. The latter obtained virus known to be pure, and used it extensively. In the course of its progress through different individuals, it must have been contaminated by its admixture in individuals affected with varioloid. Such is the belief of the physician refered to; though, at one time he did not know how to account for the strange accidents met with. A report of these cases may be found in the Virginia Med. Jour. for April, (remarkable consequences of vaccination by William W. Parker, M. D., Richmond, Va.) Dr. Parker used the precautions generally adopted by medical men, of seeing the patient on the *eighth* or *ninth* day of vaccination. His experience proves this not to be sufficient to insure genuine virus. He now finds, what he was not aware of at the time of his publication, that the individuals from whom he procured the virus producing these unfortunate consequences, had an eruption on the 12th day after vaccination, which, from the description, he believes must have been varioloid. An appendix to his cases will be published in the May number of the Virginia Medical Journal, setting forth these facts. To prevent such accidents, it is evident the medical man should see his cases more than once after vaccination; that he should not only see them on the 8th or 9th day, but on the 13th or 14th. A rule pursued by one of the old practitioners of our State, now dead, of seeing such cases every other day, was the best that could be adopted; but, unfortunately, persons at the present day do not sufficiently appreciate the advantages of genuine vaccine disease to pay the practitioner for these visits. They complain often enough now of our charge of $3.

*The conduct of the vaccine agent, in relation to the distribution of impure virus, has been investigated by the proper authorities, and he stands before the public most triumphantly acquitted of all blame.* —(See the report of the committee appointed by the legislature of Virginia, to whom this subject was referred.) In times of panic produced by the spread of small pox, it is impossible for the agent to procure virus to meet the many demands made on him, from his own vaccinations, and consequently, he is forced to send to northern cities to procure supplies, and in this way probably impure matter was imposed upon him. Virus distributed by the agent produced cases of variola and varioloid in the counties of New Kent, Caroline, and some others. Complaints were made to the Governor concerning the distribution of this impure virus; and Dr. Wm. A. Patteson, of this city, was appointed by him to repair to the county of New Kent, and examine the cases which occurred there. Dr. Patteson, after performing this duty made a report to the Governor, which was published among the documents of the Legislature, and which may be found republished in the April number of the Virginia Medical Journal. Dr. Patteson examined personally, or had communicated to him by the physicians who attended them, about forty-eight cases, forty of which were varioloid, and eight genuine variola. He concludes that the virus used was not that of small pox or varioloid, but "that it was a vaccine crust crossed from the arm of some patient who had been exposed to the contagion of small pox, in whose blood small pox was incubating when vaccined, and sufficiently matured to modify it." Upon what this supposition rests, we are not informed, unless it be that "its use (that of the virus) may produce small pox, varioloid, and apparent vaccine, the sickness of all being the direct sequitur of the use of the virus, about the usual time, and in the usual manner.

The *vaccine* disease refered to was probably that produced by matter sent by the agent to Dr. Jones of New Kent, which "proved," Dr. Patteson says, "to be *genuine vaccine.*" This same matter, he afterwards says, was given to Wm. Timberlake, who used it on fourteen members of his family, most of whom had varioloid mildly, one of them very severely. Might not these cases have been the eruption which sometimes attends vaccination; or might they not have been communicated from other cases which were prevailing in the neighborhood?* That a very decided eruption sometimes attends the vaccine disease, is well established. Wilson, p. 151, relates several cases of this kind: one of which was so severe, as to be attended with fatal effects. Eberle states, that in the report of the Central Vaccine Committee of France, for 1818-19, it is said, "that no inconsiderable number of cases occurred, in which a spontaneous eruption of many pustules appeared after vaccination, and that the matter taken from these pustules produced the disease as perfectly in others, as that taken from the primary pustule." Or, is Dr. Patteson's theory based on the fact, that so few had genuine variola after the use of the virus? The effects produced would seem just what we might expect from the use of small pox or varioloid virus, leaving out the cases referred to, resulting from the virus used by Dr. Jones. Particularly are the effects what we might expect from varioloid virus, when, too, we recollect some, if not many, of these occurred in persons previously vaccinated. These effects we might *a priori* conclude would be milder than those resulting from inoculation of small pox matter, and experiment supports this supposition—varioloid generally produces varioloid, sometimes small pox. Of course by inoculation the disease would be milder than when taken in the natural manner. I have lately been informed by a medical friend in the county of Henrico, that he saw in his family four cases of varioloid in the *unprotected*, communicated from cases of varioloid in the same house. Inoculation with genuine small pox matter produces a pustular eruption of mild character generally, running through the course of small pox, but with absence of *secondary fever*. If this regular progression through the different stages of the eruption is to be considered the characteristic

* I have since been informed that Dr. Jones obtained two different specimens of virus from the agent, though it is not so stated in the report.

of the affection, then inoculation produces small pox. If the absence of the *secondary fever* is the gist of the difference, as we believe it should be considered, then varioloid is the result. The *practical* essential of varioloid we believe to be the absence of *secondary fever*, and we see no reason for confining the term varioloid to cases in which vaccination has been previously practised. Some writers do not so confine it; and some of the old writers mention many spurious kinds of small pox prevailing before the introduction of vaccination or inoculation. They describe "vesicular, pustular and spurious small pox, swine pox, sheep pox, stone pox, horn pox," &c., all of which were regarded as having but one origin, namely, variolous contagion. In Dr. Patteson's report is mentioned a case which was reported to him by the physicians of New Kent, where a child had varioloid contracted from a person suffering with variola.

Some cases of inocculation with small pox virus, we are told, only resulted in the production of a single pustule, at the point of insertion, and why may not the apparently anomalous cases of Dr. Jones', mentioned by Dr. Patteson, be explained in this way. Two or three of these cases seemed genuine vaccination, the other fourteen mild varioloid, with one exception, which was severe.

Dr. Patteson's theory, as far as we have been able to discover from the examination of various authorities, seems unsupported by any facts. If this contamination of vaccine virus was produced by small pox incubating in the system of the vaccinated person, why have not these accidents occurred before, as they appear never to have done? Dr. Smith, formerly the United States agent for vaccination, says indeed, "that the virus of small pox intermixes with vaccine matter by a natural and unavoidable process, and in a manner that may elude the utmost care and vigilance of any person to prevent it." (American Med. Reporter, January, 1829, p. 118.) But this assertion is unsupported, and as Dr. Brown, author of the paper just referred to, in the American Med. Reporter, remarks, "this statement was probably intended to screen him from the imputation of negligence, and he will reply to it in the language of Dr. Macauly of Baltimore." "It has been known almost trom the first discovery of vaccination, that if matter be taken from a vaccine vesicle in a constitution where small pox is *manifest*, that it will in almost all cases produce the variolous disease, or some of its modifications; for by a general law of the economy, the most forcible contagion will invariably predominate, but we have in vain sought for an instance either in the records of our art, or during the prevalence of small pox in our city, where vaccine matter, taken from a subject in whose system no symptom of small pox contagion has been observed to exist, in which it has ever excited the variolous disease. If, as is plainly inferred by the circular, the contagion of small pox is capable of influencing the vaccine virus, even in systems in which small pox itself is not manifested, then there is no surety. Fortunately, no such fact has ever been remarked at the institution in London, where small pox has been more prevalent at all times since its establishment than it has been in this city (Baltimore,) of late, nor has it ever been verified by our own observations."—(American Med. Recorder loc. cit.) The supposition we have made, we believe, is the only one which will answer the facts in the case, that the virus was either that of variola or varioloid, or vaccine virus which had had imparted to it the virtues of virus of small pox or varioloid by the *manifestation* of these diseases on the subject who had been vaccinated. This latter supposition is strengthened by the facts occurring in Dr. Parker's cases, where an eruption is now known to have occurred, believed to be varioloid, in those from whom he used the vaccine scab, producing varioloid and variola. There can be no other explanation of the result he records. His matter was originally good—was used on persons who had been exposed, during the epidemic, to small pox, and in whom varioloid broke out during the progress of the vaccination, and thus contaminated the scab which was used on others. Some have doubted the change of the character of the vaccine scab by the coëxistence in the same person of varioloid; and cases are recorded where the vaccine scab produced, under these circumstances, true vaccine disease. The vaccine scab is probably frequently not changed in its nature; but Dr. Gregory, of London, vaccinated a subject in one arm, and inoculated him in the other—the diseases prevailed together, varioloid eruption occurred, and the virus of the vaccine scab, introduced in the arm of another subject, produced varioloid.—(Cyclopedia of Practical Medicine.) This experiment seems conclusive. We might very naturally expect this result, for, leaving out the very probable contamination of the vaccine scab and admixture, and interchange of properties through the circulation, we should expect that the vaccine scab might imbibe, and retain the properties of the varioloid as a piece of clothing would do.

Experiments have been instituted with the microscope, with the view of distinguishing vaccine and varioloid matter, and of determining the purity and protective properties of the former, but it would seem, without any definite results. M. Dubois (d' Amiens,) states that he has discovered, under the microscope, the distinctive characters of genuine vaccine virus. Other observers, among them M. M. Bousquet, Pelletier, Fiaid, and Donne, deny that the microscope can accomplish this desirable end.

As regards the treatment of this epidemic in Richmond, nothing new has been developed. There is reason to believe, I am afraid, that we have too much neglected treatment, at least as regards the *ectrotic* or local treatment.

Formerly, much reliance was placed on the use of mercury in the treatment of small pox.

Good ("Study of Medicine,") says, mercury appears to have a specific influence upon the action of variolous matter —for though, when considerably diluted with water, it is still capable of propagating the disease by inocculation, yet Von Wensel has shown satisfactorily, that when triturated with calomel it loses its energy, and in inoculation, becomes inert." Experience in hospitals, he also says, shows the disease to be particularly mild in those patients who happened to receive the infection soon after ptyalism. These ideas may be considered exploded, nevertheless it is equally probable, we are too much in the habit of looking upon eruptive diseases as prone to have their own way, and as not amenable to treatment.

A strong, robust negro man fell under my treatment during the recent epidemic, with the following symptoms: high fever, injected eyes, much headache and backache, with hot skin, and furred tongue. The bowels were constipated, and he was ordered 12 grs. comp. ext. col. and 12 grs. submur. hyd. I

suspected varioloid, as I knew he had been recently exposed to varioloid by being with his brother, who, at the time, was suffering with it. The bowels, by the next day, were freely moved, and all the symptoms much mitigated. The night following the action of the purgative, the eruption of varioloid commenced, and proved to be very mild—much contrary to my expectations and fears. He had been vaccinated when a child, as his brother had been. Purgatives are highly recommended by the French authors, in the beginning of the disease, when the symptoms of fever run high. Watson says, "you may abate the force of the eruptive fever, and keep down, it is believed, the number of pustules, by saline purgatives, so exhibited as to produce two or three loose stools every day, and free ventilation of the surface of the body.

At the "hospital," I saw tinct. iodine tried in one case of severe confluent small pox, as a local application to the face, to prevent the pitting, with considerable effect. "The employment of the plaster of Vigo *cum mercuris*, as an abortive treatment, was laid down certainly by Zimmerman. It has more recently been extolled by MM. Gariel, Nonat, and especially by M. Briquet, who has given to it a very extensive use. The experiences of these observers which we have frequently repeated, have demonstrated to us the powerful action of topical mercurial applications upon varioloid eruptions. The application of the plaster of vigo of the codex, is preferable to that of mercurial ointment. It sometimes brings about the resolution of the variolous eruption, and oftener converts it into vesicles. It should not be delayed to the fifth or sixth day, but the sooner it is had recourse to, the more certain is the effect. An application of four or five days is sufficient." —(Dictionnaire de Medecine, Tome 30, p. 582.)

Wilson, (Disease of the Skin,) extols the mercurial application. He says that Dr. Oliffe has made it the subject of an essay, read before the Parisian Medical Society. "Mercury administered internally," he says, "has long been known to possess remarkable powers in modifying the influence of variola upon the system, but it was left for modern times to prove that this agent has also the property of neutralizing the variolous poison, when applied externally." He relates cases where the effect was very striking, applied on the second or third day at least of the eruption. He gives the formula for its preparation, (p. 131,) by the French Pharmacopeia.

Dr. Hughes Bennet, of Edinburgh, adopted with success the mercurial ectrolic treatment—he used ung. hydrarg. fort. ʒj, with powdered starch, ℥j. The ointment was applied pretty thickly, night and morning, over the face, with the result of preventing itching, and swelling of the skin, and deep red stains, which small pox commonly leaves behind it, and the formation of pits.

For the same pupose, nitrate of silver has been used by MM. Serres, Bretonneau and Velpeau, and they say, with success. Besides the pain and fever it produces, the testimony in its favor by no means compares with that of the mercurial application. Great benefit is said to arise from pursuing the practice of the Arabian physicians, of opening the pustules when matured, gently pressing out their contents and wiping it away with a sponge wetted with water, or an infusion of poppies. It is said to prevent the formation of deep and disfiguring cicatrices, and promote the healing of the ulcerations. —(Wilson on the Skin, p. 130.)

