# Errata

**Published:** 1856-07

**Authors:** 


### ERRATA.

To June number, Article 1. By Thos. Pollard, M. D.:

On page 579, in 7th line from bottom, for *long* read *being*—on same page, in 8th line from top, for *vaccinated* read *re-vaccinated.* On page 584, in 7th line from top, for *head* read *breast.* On page 585, in 8th line from bottom, for *crossed* read *cropped.* On page 587, 14th line from top place *unprotected* before *child.* On page 590, for *Vigo cum Mercuris* read *Vigo cum Mercurio.* On page 591, for *Ectrolic* read *Ectrotic.*

